# Supplementary material for: Potential of Enzymatically Synthesized Hemozoin Analog as Th1 Cell Adjuvant
Source: Nanomaterials (Basel). 2024 Sep 3;14(17):1440. doi: 10.3390/nano14171440 (PMC11397214; doi:10.3390/nano14171440)
Supplement: Supplementary file 1 [file nanomaterials-14-01440-s001.zip › nanomaterials-3122701-supplementary.pdf]

Supporting Information

Potential of Enzymatically Synthesized Hemozoin Analog as Th1 Cell Adjuvant

Kazuaki Hoshi [1], Anh Thi Tram Tu [1,2,3], Miwako Shobo [1], Karin Kettisen [4], Lei Ye [4], Leif Bülow [4,*], Yoji Hakamata [5], Tetsuya Furuya [6], Ryutaro Asano [7], Wakako Tsugawa [7], Kazunori Ikebukuro [7], Koji Sode [8] and Tomohiko Yamazaki [1,9,*]

1 Research Center for Macromolecules and Biomaterials, National Institute for Materials Science (NIMS), Tsukuba 305-0047, Japan; k.hoshi.oe@juntendo.ac.jp (K.H.); tttanh@hcmus.edu.vn (A.T.T.T.); shobo.miwako@nims.go.jp (M.S.)
2 Department of Magnetic and Biomedical Materials, Faculty of Materials Science and Technology, University of Science, Ho Chi Minh City 70000, Vietnam
3 Ho Chi Minh City Campus, Vietnam National University, Thu Duc City, Ho Chi Minh City 70000, Vietnam
4 Division of Pure and Applied Biochemistry, Department of Chemistry, Lund University, 22100 Lund, Sweden; karinkettisen@gmail.com (K.K.); lei.ye@tbiokem.lth.se (L.Y.)
5 School of Veterinary Nursing and Technology, Nippon Veterinary and Life Science University, Musashino 180-8602, Tokyo, Japan; yhakama@nvlu.ac.jp
6 Cooperative Department of Veterinary Medicine, Faculty of Agriculture, Tokyo University of Agriculture and Technology, Fuchu 183-8509, Tokyo, Japan; furuyat@cc.tuat.ac.jp
7 Department of Biotechnology and Life science, Graduate School of Engineering, Tokyo University of Agriculture and Technology, Koganei 184-8588, Tokyo, Japan; ryutaroa@cc.tuat.ac.jp (R.A.); tsugawa@cc.tuat.ac.jp (W.T.); ikebu@cc.tuat.ac.jp (K.I.)
8 Joint Department of Biomedical Engineering, The University of North Carolina at Chapel Hill and North Carolina State University, Chapel Hill, NC 27599, USA; ksode@email.unc.edu
9 Graduate School of Life Science, Hokkaido University, Sapporo 060-0808, Japan
* Correspondence: yamazaki.tomohiko@nims.go.jp (T.Y.); leif.bulow@kilu.lu.se (L.B.)

Supplementary Figures 1-2

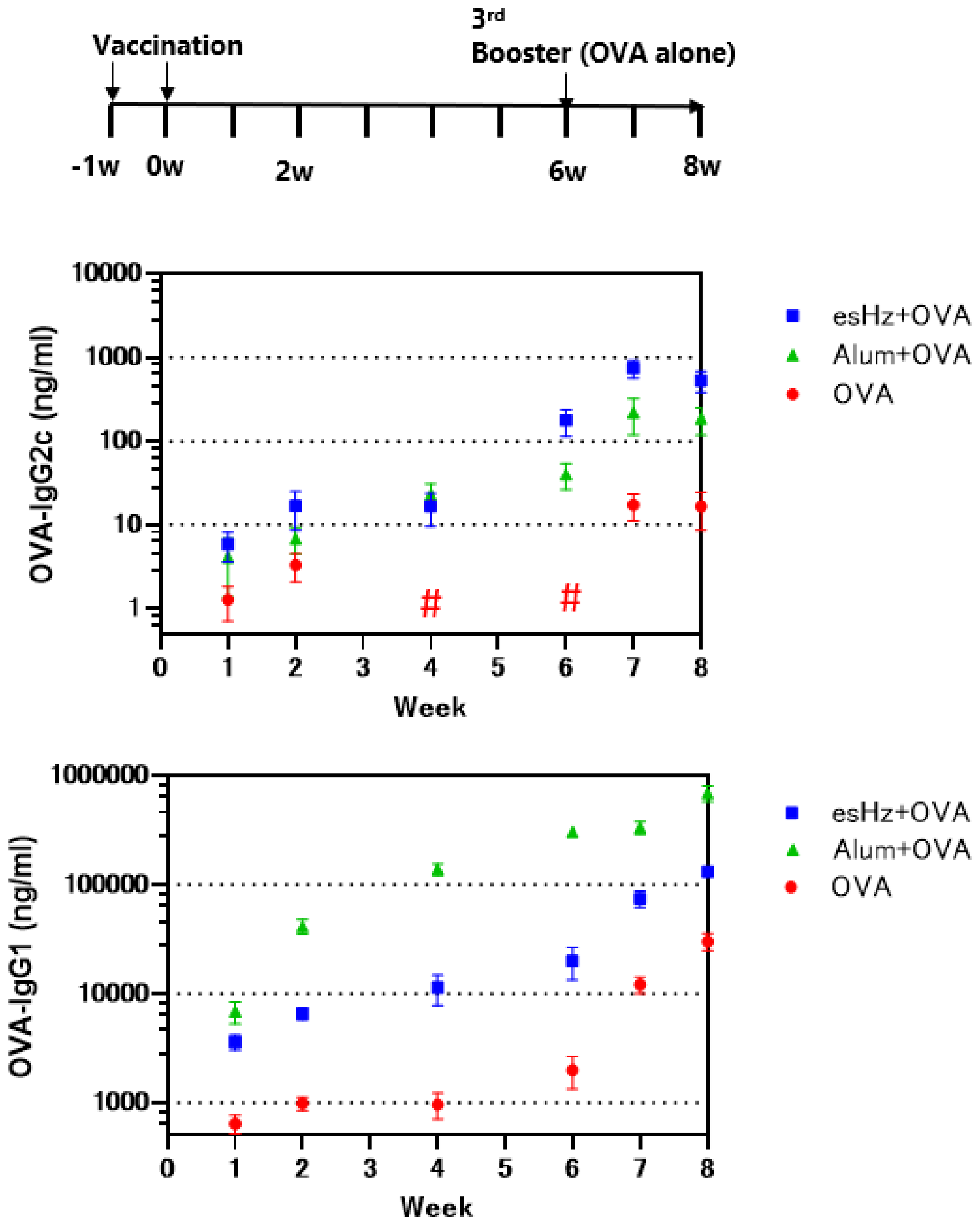

Figure S1. Changes in ovalbumin (OVA)-specific IgG2c and IgG1 levels in mouse serum after the second subcutaneous dose.

Serum OVA-specific IgG2c and IgG1 levels were measured using enzyme-linked immunosorbent assays. Mice were immunized with OVA (200 μg) and an enzymatically synthesized analog of hemozoin (esHz) (500 μg), OVA (200 μg), alum (500 μg), and OVA alone at one-week intervals, and the mice were boosted with OVA (200 μg) at six weeks after the second dose. Results are expressed as mean ± standard error of the mean (n = 10 to 12). #The levels of OVA-specific IgG2c were lower than the detectable minimum of 0.39 ng/mL.

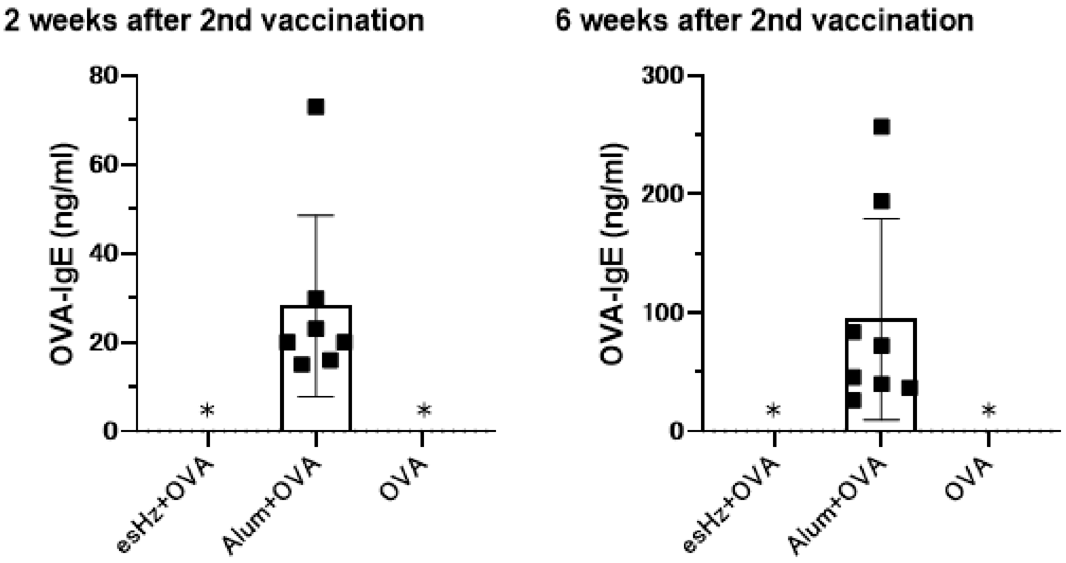

Figure S2. Alum induces ovalbumin (OVA)-specific IgE but not the enzymatically synthesized analog of hemozoin (esHz).

OVA-specific IgE levels were induced by either the esHz or alum adjuvant at two and six weeks after the second dose of the vaccine. Serum collected from the immunized mice (Figure 5) was used for OVA-specific IgE measurement using enzyme-linked immunosorbent assay. Results are expressed as the mean ± standard deviation (n = 8). * OVA-IgE levels were lower than the detectable minimum of 15 ng/mL.
